# Chloride and sulfate salinity differently affect biomass, mineral nutrient composition and expression of sulfate transport and assimilation genes in *Brassica rapa*

**DOI:** 10.1007/s11104-016-3026-7

**Published:** 2016-08-25

**Authors:** Martin Reich, Tahereh Aghajanzadeh, Juliane Helm, Saroj Parmar, Malcolm J. Hawkesford, Luit J. De Kok

**Affiliations:** 1 Laboratory of Plant Physiology, Groningen Institute for Evolutionary Life Sciences, University of Groningen, P.O. Box 11103, 9700 CC Groningen, The Netherlands; 2 Department of Biology, Faculty of Basic Science, University of Mazandaran, Babolsar, Iran; 3 Plant Biodiversity Group, Institute of Systematic Botany, Friedrich Schiller University, Philosophenweg 16, D-07743 Jena, Germany; 4 Department of Plant Biology and Crop Science, Rothamsted Research, Harpenden, Herts AL5 2JQ UK

**Keywords:** Salt stress, Sodium, Potassium, Sulfate uptake, Vacuolar sulfate transport, Sulfur assimilatory enzymes

## Abstract

**Background and aims:**

It remains uncertain whether a higher toxicity of either NaCl or Na_2_SO_4_ in plants is due to an altered toxicity of sodium or a different toxicity of the anions. The aim of this study was to determine the contributions of sodium and the two anions to the different toxicities of chloride and sulfate salinity. The effects of the different salts on physiological parameters, mineral nutrient composition and expression of genes of sulfate transport and assimilation were studied.

**Methods:**

Seedlings of *Brassica rapa* L. have been exposed to NaCl, Na_2_SO_4_, KCl and K_2_SO_4_ to assess the potential synergistic effect of the anions with the toxic cation sodium, as well as their separate toxicities if accompanied by the non-toxic cation potassium. Biomass production, stomatal resistance and *Fv/fm* were measured to determine differences in ionic and osmotic stress caused by the salts. Anion content (HPLC), mineral nutrient composition (ICP-AES) and gene expression of sulfate transporters and sulfur assimilatory enzymes (real-time qPCR) were analyzed.

**Results:**

Na_2_SO_4_ impeded growth to a higher extent than NaCl and was the only salt to decrease *Fv/fm*. K_2_SO_4_ reduced plant growth more than NaCl. Analysis of mineral nutrient contents of plant tissue revealed that differences in sodium accumulation could not explain the increased toxicity of sulfate over chloride salts. Shoot contents of calcium, manganese and phosphorus were decreased more strongly by exposure to Na_2_SO_4_ than by NaCl. The expression levels of genes encoding proteins for sulfate transport and assimilation were differently affected by the different salts. While gene expression of primary sulfate uptake at roots was down-regulated upon exposure to sulfate salts, presumably to prevent an excessive uptake, genes encoding for the vacuolar sulfate transporter Sultr4;1 were upregulated. Gene expression of ATP sulfurylase was hardly affected by salinity in shoot and roots, the transcript level of 5′-adenylylsulfate reductase (APR) was decreased upon exposure to sulfate salts in roots. Sulfite reductase was decreased in the shoot by all salts similarly and remained unaffected in roots.

**Conclusions:**

The higher toxicity of Na_2_SO_4_ over NaCl in *B. rapa* seemed to be due to an increased toxicity of sulfate over chloride, as indicated by the higher toxicity of K_2_SO_4_ over KCl. Thus, toxicity of sodium was not promoted by sulfate. The observed stronger negative effect on the tissue contents of calcium, manganese and phosphorus could contribute to the increased toxicity of sulfate over chloride. The upregulation of Sultr4;1 and 4;2 under sulfate salinity might lead to a detrimental efflux of stored sulfate from the vacuole into the cytosol and the chloroplasts. It remains unclear why expression of Sultr4;1 and 4;2 was upregulated. A possible explanation is a control of the gene expression of these transporters by the sulfate gradient across the tonoplast.

## Introduction

Soil salinity is a threat to agricultural production and a better understanding of the physiological basis underlying salt stress will be essential to improve the salt tolerance of crop plants (Flowers and Yeo 1995; Chinnusamy et al. 2005; Ashraf et al. 2008). Most studies on salt stress and tolerance have been conducted with NaCl, which is generally the most abundant salt in saline soils. An excess of sulfate salts may occur in volcanic soils, in marine soils (as sea water contains high amounts of sulfate), in agricultural soils irrigated with saline water or may be caused by anthropogenic inputs from industry via wet or dry deposition of atmospheric sulfur gases (Moss 1978; Nriagu 1978; Freedman and Hutchinson 1980; Chang et al. 1983).

The negative effects of NaCl salinity on plant growth and performance are generally attributed to the toxicity of Na^+^, which at high cytosolic concentrations may disrupt K^+^ homeostasis, negatively affecting functioning of a large group of enzymes (Pardo and Quintero 2002; Zhu 2007; Kronzucker and Britto 2011). Furthermore, it has been observed that in some species, chloride may be more toxic than Na^+^ (White and Broadley 2001). Moreover, there are differences in the susceptibility of plant species to NaCl and Na_2_SO_4_ salinity: Na_2_SO_4_ was shown to be more toxic than NaCl in wheat (Datta et al. 1995), sugar cane (Joshi and Naik 1980), sugar beet and tomato (Eaton 1942), wild potato (Bilski et al. 1988), barley (Huang and Redmann 1995), in calli of *Brassica campestris* (Paek et al. 1988) and for the germination of alfalfa (Redmann 1974) and wheat (Hampson and Simpson 1990). The halophyte *Prosopis strombulifera,* which tolerates high concentrations of NaCl, was shown to be still sensitive to Na_2_SO_4_ (Reginato et al. 2014).

The physiological basis of sulfate toxicity is poorly understood. Sulfate taken up by the root is in general the major sulfur source for plant growth. It is reduced in the chloroplasts (and in plastids in the root) to sulfide prior to its assimilation into cysteine, which is the precursor and/or reduced sulfur donor for the majority of the other organic sulfur compounds in plants (Hell et al. 2002; Saito 2004; Hawkesford and De Kok 2006). The uptake of sulfate by the root, its distribution in plants and its reduction in the chloroplast/plastids is under strict regulatory control and adjusted to the sulfur demand for growth (Hawkesford and De Kok 2006; Koralewska et al. 2007, 2008, Koralewska et al. 2009). Several intermediates of the sulfur reduction pathway are toxic (Cerović et al. 1982; Rennenberg 1984), but also sulfate itself was reported to have toxic effects on plant metabolism via an inhibition of photophosphorylation (Ryrie and Jagendorf 1971). If, for instance, Chinese cabbage was exposed to ≥20 mM Na_2_SO_4_, not only was plant biomass production affected, but also the regulation of the uptake and assimilation of sulfate (Reich et al. 2015). This resulted in a downregulation of the expression and activity of the sulfate transporters and in a downregulation of the expression of 5′-adenylylsulfate reductase (APR) (one of the key regulating enzymes in the sulfate reduction pathway) in the root.

An understanding of the contribution of sulfate to the toxicity of soil salinity will be important to understand and increase salt tolerance of crops under conditions in which not only NaCl, but additionally sulfate salts are also present. The aim of the present study was to determine whether a higher toxicity of either NaCl or Na_2_SO_4_ in *Brassica rapa* was due to an altered toxicity of sodium or different toxicities of the anions. Plants were exposed to either NaCl or Na_2_SO_4_ salinity at equimolar sodium concentrations, and additionally, another set of plants was exposed to either KCl or K_2_SO_4_ with the aim to separate the toxicity of sodium from the toxicity of chloride or sulfate. Biomass production, stomatal resistance and maximum quantum efficiency of photosystem II (*Fv/fm*) were measured to assess the toxicity of the different salts in *B. rapa*. Anion contents were measured to investigate possible differences in accumulation of sulfate and chloride, as well as to compare their impact on nitrate content. Mineral nutrient composition was analyzed to explore possible differences in the effect on other essential nutrients and to test if there is a difference in the accumulation of sodium. Finally, the effects of chloride and sulfate salinity on the uptake, vacuolar storage and reduction of sulfate was studied by measuring the expression of Group 1 sulfate transporters, which are responsible for primary sulfate uptake (Smith et al. 1995; Hawkesford and De Kok 2006), Group 4 sulfate transporters which are responsible for the vacuolar exchange of sulfate (Kataoka et al. 2004b; Hawkesford and De Kok 2006), and enzymes of the sulfur reduction pathway.

## Material and methods

### Plant material and growing conditions

Seeds of *Brassica rapa*, cv. Komatsuna (Van der Wal, Hoogeveen, The Netherlands) were germinated in vermiculite. Ten day-old seedlings were transferred into a 25 % Hoagland nutrient solution (pH 5.9) consisting of 1.25 mM Ca(NO_3_)_2_ × 4 H_2_O, 1.25 mM KNO_3_, 0.25 mM KH_2_PO_4_, 0.5 mM MgSO_4_ × 7 H_2_O, 11.6 μM H_3_BO_3_, 2.4 μM MnCl_2_ × 4 H_2_O, 0.24 μM ZnSO_4_ × 7 H_2_O, 0.08 μM CuSO_4_ × 5 H_2_O, 0.13 μM Na_2_MoO_4_ × 2 H_2_O and 22.5 μM Fe^3+^-EDTA) in 30 l containers (20 sets per container, three plants per set) which were placed in a climate controlled room for the duration of the experiment. Relative humidity was 60–70 % and the photoperiod was 14 h at a photon fluence rate of 300 ± 20 μmol m^−2^ s^−1^ (within the 400–700 nm range) at plant height, supplied by Philips GreenPower LED lamps (deep white/red 120). Day/night temperatures were 21/18 °C. Ten-day old seedlings were grown without additional salt for three days and subsequently salt concentrations were gradually increased during the following three days. For NaCl and KCl the steps were 25, 50 and 100 mM and for Na_2_SO_4_ and K_2_SO_4_ the steps were 12.5, 25 and 50 mM. For half of the plants the final concentrations were 50 mM for the chloride and 25 mM for the sulfate salts, for the other half 100 mM for the chloride and 50 mM for the sulfate salts. Seedlings were grown in the final concentrations for nine and eight more days, respectively, and were harvested at day 21 after sowing. *Fv/fm*, stomatal conductance, growth and anion content were determined from both concentrations. Mineral nutrient composition and gene expression were only determined for the high salt concentrations.

### Harvest and growth analysis

Plants were harvested at the end of the experiment and shoot and roots were separated and their fresh weight was determined immediately. For determination of the dry matter content fresh plant tissue was dried at 80 °C for 24 h and stored in a desiccator for further use. Fresh material was stored in either −20 °C or −80 °C, depending on the requirements for further analysis.

### Stomatal resistance and maximum quantum efficiency of photosystem II (*Fv/fm*)

Prior to harvest, *Fv/fm* of leaves in dark adapted conditions was determined in the morning before light was switched on (PAM 2000, Walz, Effeltrich, Germany). After the light had been switched on for several hours, stomatal resistance was measured on the underside of the largest leaf (AP4 Leaf Porometer, Delta-T Devices Ltd., Cambridge, UK).

### Anion determination

Anions were extracted from frozen plant material in water and determined refractometrically after separation by HPLC (Aghajanzadeh et al. 2014).

### Mineral nutrient composition

For the determination of mineral nutrient contents, dried leaf tissues (0.2–0.5 g) were digested with 5 ml of nitric acid: perchloric acid (87:13, *v*/v; 70 % concentration, trace analysis grade; Fisher Scientific; Zhao et al. 1994). The minerals in the digested samples were analyzed by inductively coupled plasma atomic emission spectrometry (ICP-AES) analysis. Repeat samples were carried out every 10 samples; blanks and standard reference material (NIST 1567, a wheat flour) were used for quality control. The sample introduction system consisted of a micromist glass concentric nebulizer, quartz Scott-type double-pass spray chamber at 2 °C, and nickel sample (1 mm) and skimmer (0.4 mm cones). Operating parameters were optimized daily using a tune solution containing 1 μg l^−1^ cerium, lithium, tellurium, and yttrium. Other instrument conditions were radiofrequency forward power of 1550, sample depth of 8.0 mm, carrier gas flow rate of 0.89 l min^−1^, reaction gas flow rate of 4 ml min^−1^ (H_2_) or of 4.5 ml min^−1^ (helium). An internal standard (500 μg l^−1^ germanium) was used to correct for signal drift. Mineral nutrient contents were measured in dried material. These contents were multiplied with the average dry matter content to calculate the contents based on fresh weight.

### RNA extraction

Total RNA was isolated by a modified hot phenol method (Verwoerd et al. 1989). Frozen ground plant material was extracted in hot (80 °C) phenol/extraction buffer (1:1, *v*/v), 1 g ml^−1^. The extraction buffer contained 0.1 M Tris–HCl, 0.1 M LiCl, 1 % SDS (*w*/*v*), 10 mM EDTA, pH 8.0). After mixing, 0.5 ml of chloroform–isoamyl alcohol (24:1, v/v) was added. After centrifugation (13,400×g) for 5 min at 4 °C, the aqueous phases were transferred to a new tube. After adding an equal volume of chloroform and isoamyl alcohol, the total RNA was precipitated by 4 M LiCl overnight at 4 °C. Total RNA was collected and washed with 70 % ethanol. Possible genomic DNA contamination was removed with a DNAase treatment step (Promega, USA). Phenol–chloroform–isoamyl alcohol and chloroform–isoamyl alcohol were used for further purification and total RNA was precipitated by ethanol and dissolved in diethylpyrocarbonate-treated water. The quantity and quality of RNA was checked using ThermoNanoDrop 2000 and RNA in each was adjusted to the same concentration. The integrity of RNA was verified by electrophoresis by loading 1 μg RNA on a 1 % TAE-agarose gel.

### Real-time quantitative PCR of genes of group 1 and group 4 sulfate transporters and of sulfur assimilatory enzymes

DNA-free intact RNA (1 μg) was reverse transcribed into cDNA with oligo-dT primers using a first strand cDNA synthesis kit (Promega, USA) according to the manufacture-supplied instructions. Subsequently, the cDNA was used as a template in real-time PCR experiments with gene-specific primers. To design primers for genes involved in sulfate uptake, vacuolar remobilization and reduction, the CDS of *Arabidopsis thaliana* genes were used to query homologous *B. rapa* sequences which are available in the *B. rapa* genome sequence portal http://brassicadb.org/brad/. The full length sequences of these genes can be found under following accession numbers: Sulfur transporter 1.1 (Sultr1;1 XM009128953), Sulfur transporter 1.2 (Sultr1;1 XM009108197, XM009108195 and XM009108196), Sulfur transporter 4.1 (Sultr4;1 XM009123507 and NM121358.2), Sulfur transporter 4.2 (Sultr4;2 XM009136985 and NM112087.2), ATP sulfurylase (ATPS XM009147003, XM009150169, XM009103518 and XM009151241) APS reductase (APR XM009116311, XM009138987 and XM009125111), sulfite reductase (SiR (XM009136868, XM009124516 and XM009124333). Relative transcript levels were normalized based on expression of *Arabidopsis thaliana* actin 2 gene (*ACT2)* as a reference gene. To design primers, *Arabidopsis ACT2* genes (NM_112764.3) were used to query homologous *B. rapa* (JN120480.1) sequences. Gene-specific primer sets are listed in Table 1. RT-PCR was performed on Applied Bio Systems’ 7300 real-time PCR system using the SYBR Green master mix kit (Thermo Scientific) based on manufacturer’s instructions. The relative expression was calculated based on the comparative cycle threshold (Ct) method by subtracting the Ct of the target gene from the Ct of the actin 2 gene (internal control gene). The Ct is the time at which fluorescence intensity is greater than background fluorescence (Wong and Medrano 2005). The transcript level of the target gene and actin was measured using the comparative Ct method. Analysis of qPCR data was performed using three independent RNA preparations from separate plant tissue.

**Table 1:** The list of primer sequences of forward and reverse strand of genes involved in sulfate uptake, vacuolar remobilization and reduction and the reference gene *ACT2*

	Primer sequences (5′-3′)	
Gene	Forward	Reverse
Sultr1;1	TGGCCATAGTGGTAGCTC	AGACCAAGGACGGCTGAT
Sultr1;2	GCAACAGACGGTGGAGAT	GCCCCCAATCGAAAACC
Sultr4;1	GAGGAGGTTTGGGAATAACG	AATCGCAACCCACTATACAC
Sultr4;2	CTCTCTGGCACTACGTTTG	AATAGCCGGAGAAGAAGAAG
ATPS	TTYGCKTTCCAGCTWAGG	AGGGTTTTTGWATCCCATCTC
APR	GTATGTTTCWATWGGGTGTGAG	CTYCTTGATGTTCCCTTTGTG
SiR	ATGGCTTGTCCAGCTTTTC	AGACCAACCTTCTCAAACATT
ACT2	AGCAGCATGAAGATCAAGGT	GCTGAGGGATGCAAGGATAG

### Statistical analysis

Statistical analyses were performed using GraphPad Prism (GraphPad Software Inc., San Diego, CA, USA). A one-way analysis of variance (ANOVA) was performed and the treatment means were compared using Tukey’s HSD all-pairwise comparisons at the *p* = 0.05 level as a post-hoc test (see figures).

## Results

### Biomass production

**Fig. 1:**
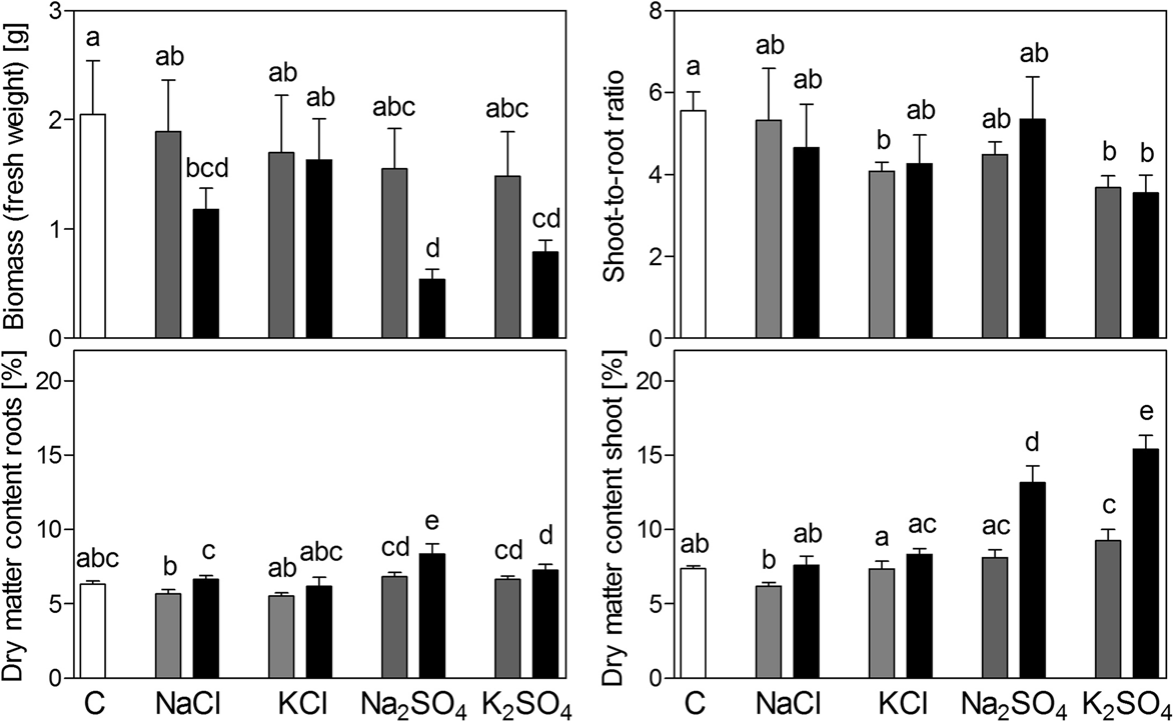
Biomass, shoot to root ratio and dry matter content of shoot and roots of *Brassica rapa* seedlings exposed to two levels of NaCl, KCl, Na_2_SO_4_ and K_2_SO_4_. Low concentrations were 50 mM for NaCl and KCl and 25 mM for Na_2_SO_4_ and K_2_SO_4_ (*grey bars*). High concentrations were 100 mM for NaCl and KCl and 50 mM for Na_2_SO_4_ and K_2_SO_4_ (*black bars*). Data represent the mean of five measurements with three plants in each (± SD). Different letters indicate significant difference (*p* < 0.05; One-way ANOVA, Tukey’s HSD all-pairwise comparisons as a post-hoc test)

Total biomass was not affected by 50 mM NaCl and KCl, 25 mM Na_2_SO_4_ and K_2_SO_4_ and 100 mM KCl (Fig. 1). 100 mM NaCl caused a reduction in total biomass of 50 %, 50 mM Na_2_SO_4_ a reduction of 74 % and 50 mM K_2_SO_4_ a reduction of 62 %. Shoot-to-root ratio was reduced by 50 mM KCl and both concentrations of K_2_SO_4_ (Fig. 1). Shoot dry matter content was slightly increased by 25 mM K_2_SO_4_ and approximately doubled by 50 mM K_2_SO_4_ and Na_2_SO_4_ (Fig. 1). Dry matter content in roots was slightly increased by 50 mM K_2_SO_4_ and Na_2_SO_4_ (Fig. 1).

### Stomatal resistance and maximum quantum efficiency of photosystem II (*Fv/fm*)

**Fig. 2:**
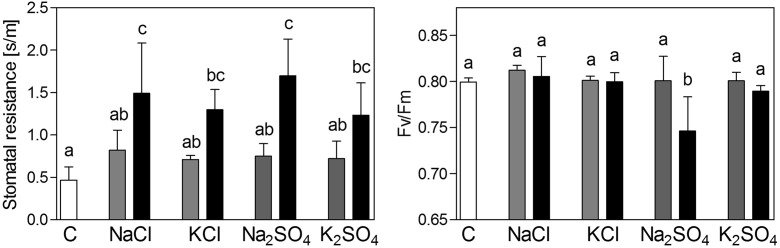
Stomatal resistance and *Fv/fm* of *Brassica rapa* seedlings exposed to two levels of NaCl, KCl, Na_2_SO_4_ and K_2_SO_4_. Low concentrations were 50 mM for NaCl and KCl and 25 mM for Na_2_SO_4_ and K_2_SO_4_ (grey bars). High concentrations were 100 mM for NaCl and KCl and 50 mM for Na_2_SO_4_ and K_2_SO_4_ (black bars). Data represent the mean of five measurements (± SD). Different letters indicate significant difference (p < 0.05; One-way ANOVA, Tukey’s HSD all-pairwise comparisons as a post-hoc test)

Stomatal resistance was increased by all salts similarly (Fig. 2) with values being approximately three-fold higher upon exposure to 100 mM NaCl and KCl and 50 mM Na_2_SO_4_ and K_2_SO_4_. *Fv/fm* was only significantly reduced by 50 mM Na_2_SO_4_ (Fig. 2).

### Anion content

**Fig. 3:**
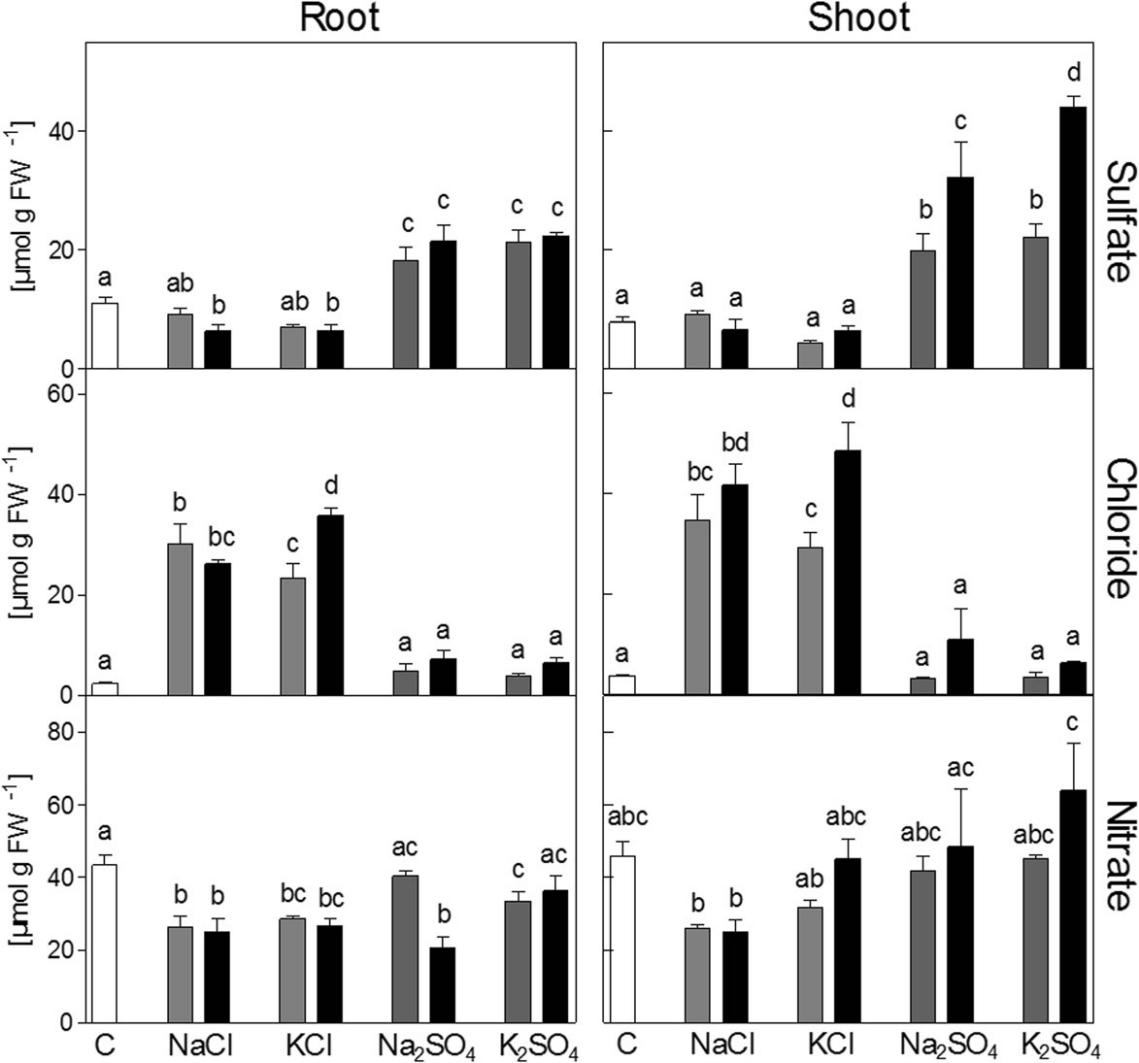
Anion content of shoot and roots of *Brassica rapa* seedlings exposed to two levels of NaCl, KCl, Na_2_SO_4_ and K_2_SO_4_. Low concentrations were 50 mM for NaCl and KCl and 25 mM for Na_2_SO_4_ and K_2_SO_4_ (grey bars). High concentrations were 100 mM for NaCl and KCl and 50 mM for Na_2_SO_4_ and K_2_SO_4_ (black bars). Data represent the mean of four measurements with three plants in each (± SD). Different letters indicate significant difference (p < 0.05; One-way ANOVA, Tukey’s HSD all-pairwise comparisons as a post-hoc test)

Plants in all treatments showed increased tissue contents of the anion supplied in excess (Fig. 3). Chloride contents remained unaffected by exposure to sulfate salts, but the sulfate content in roots was significantly decreased by 100 mM NaCl and KCl. Chloride content did not significantly increase further in plants exposed to NaCl when external concentrations were doubled, while with KCl a further increase of 30 % was observed in both shoot and roots. Sulfate content in the shoot of plants exposed to Na_2_SO_4_ and K_2_SO_4_ almost doubled, when the concentration of the salts in the medium was doubled from 25 to 50 mM. In contrast, sulfate levels in the roots remained at the same level (Fig. 3). Nitrate content in the shoot was highly variable, but addition of sulfate salts did not result in significant changes (Fig. 3). Nitrate levels in shoots were only significantly affected by 50 and 100 mM NaCl (if tested separately against the control with an unpaired Student’s t-test; not shown) which led to a decrease of 50 %. In the roots, all chloride salts and 50 mM Na_2_SO_4_ decreased nitrate content significantly (Fig. 3).

### Mineral nutrient content

**Fig. 4:**
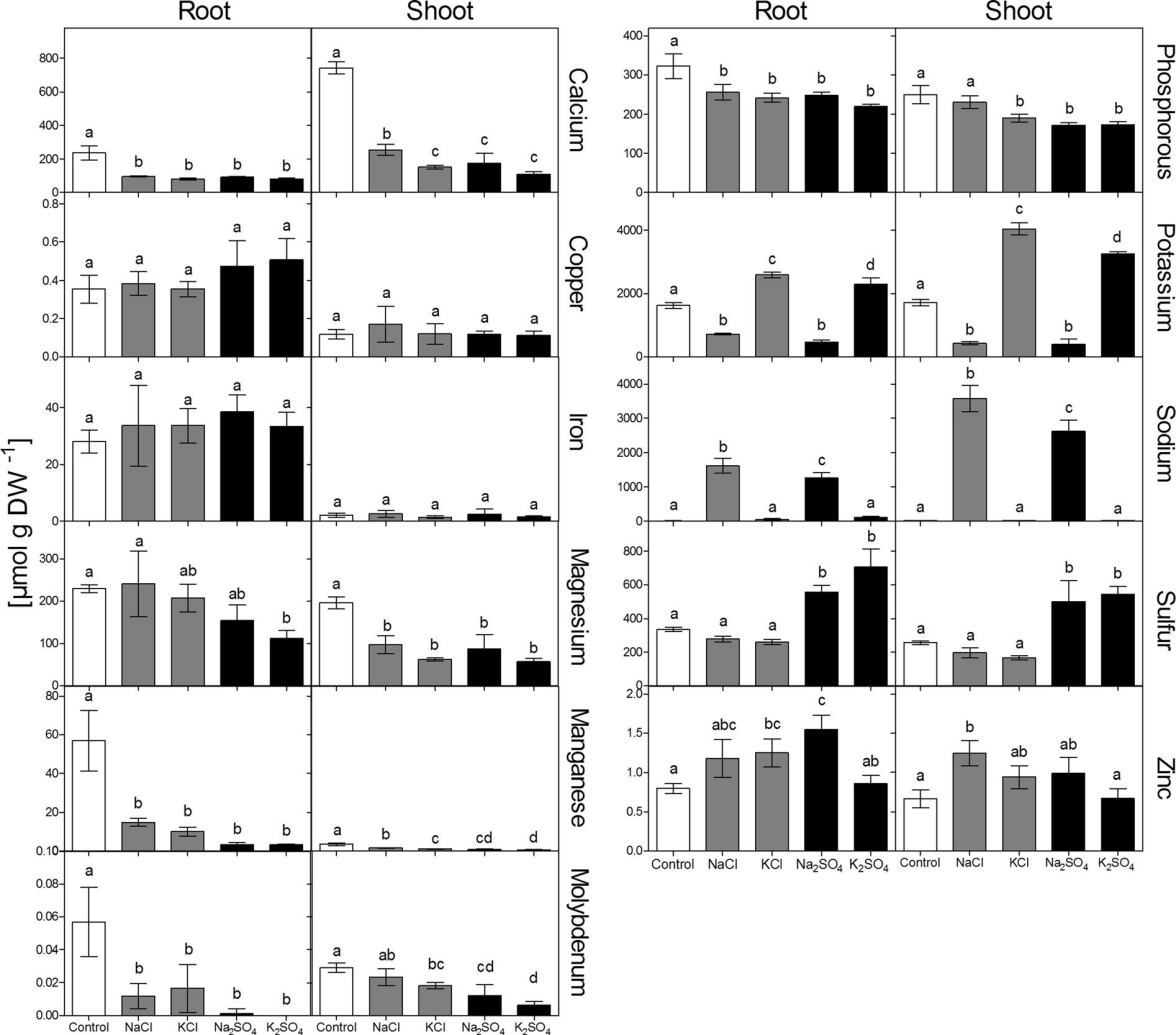
Mineral nutrient composition of shoot and roots of *Brassica rapa* seedlings exposed to two levels of NaCl, KCl, Na_2_SO_4_ and K_2_SO_4_. Concentrations were 100 mM for NaCl and KCl (grey bars) and 50 mM for Na_2_SO_4_ and K_2_SO_4_ (black bars). Data represent the mean of five measurements with three plants in each (± SD). Different letters indicate significant difference (p < 0.05; One-way ANOVA, Tukey’s HSD all-pairwise comparisons as a post-hoc test); DW = Dry weight

In the shoot, exposure to NaCl led to a slightly higher increase of sodium content than exposure to Na_2_SO_4_ (Fig. 4). Sulfur content was increased upon exposure to the sulfate salts, while chloride salts had no significant effect. Exposure to both sodium salts strongly decreased potassium content in shoot and roots to a similar extent. The same was true for calcium which was decreased slightly more by sulfate salts in the shoot. Overall, these differences were minor and potassium/sodium as well as calcium/sodium ratios were strongly but similarly decreased by NaCl and Na_2_SO_4_. The decrease of magnesium content was less pronounced than for potassium and calcium. In roots, a significant decrease was only observed upon exposure to K_2_SO_4_. In the shoot, magnesium was decreased by all salts similarly. Phosphorus content was decreased by all salts in roots and by all salts, except NaCl, in the shoot. Na_2_SO_4_ had a significantly stronger effect on phosphorus in the shoot than NaCl. The measured micronutrients were differently affected by the salts: copper and iron contents remained rather unaffected, but manganese was strongly decreased by all salts in shoot and roots, with a stronger effect of sulfate salts in roots and a stronger effect of Na_2_SO_4_ compared to NaCl in the shoot. Molybdenum content was decreased significantly in roots and shoot by all salts (except of NaCl in the shoot), with an overall stronger effect of sulfate salts. Zinc contents were significantly increased by Na_2_SO_4_ in roots and by NaCl in the shoot.

### Transcript levels of the sulfate transporters and sulfur assimilatory enzymes

**Fig. 5:**
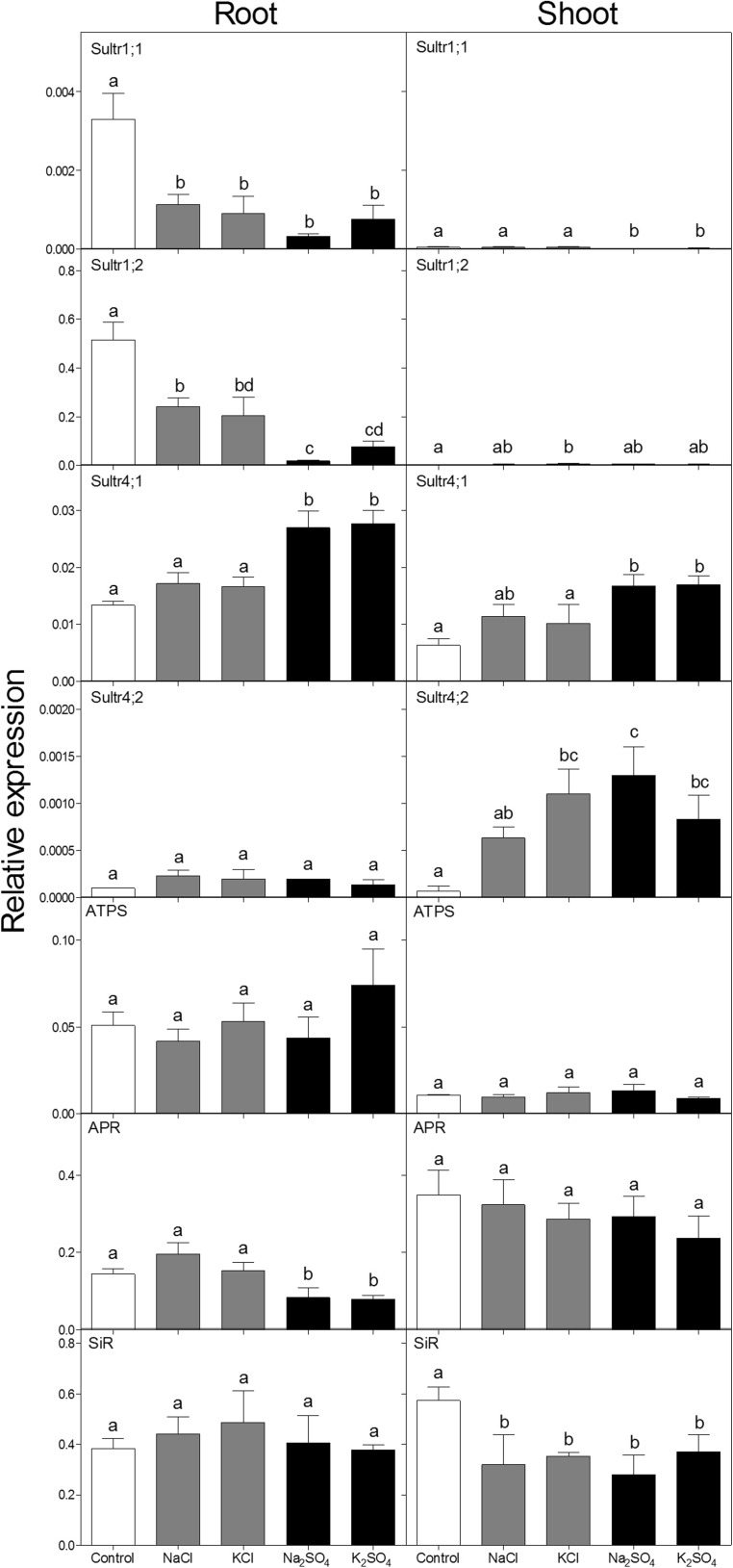
Relative gene expression of the sulfate transporters and sulfur assimilatory enzymes in shoot and roots of *Brassica rapa* sesedlings exposed to two levels of NaCl, KCl, Na_2_SO_4_ and K_2_SO_4_. Concentrations were 100 mM for NaCl and KCl (grey bars) and 50 mM for Na_2_SO_4_ and K_2_SO_4_ (black bars). Data represent the mean of three measurements with three plants in each (± SD). Different letters indicate significant difference (p < 0.05; One-way ANOVA, Tukey’s HSD all-pairwise comparisons as a post-hoc test). ATPS = ATP sulfurylase; APR = adenosine 5′-phosphosulfate reductase; SiR = Sulfite reductase

Sultr1;2 was the primary sulfate transporter expressed in the roots, whereas the expression of Sultr1;1 was very low. The expression of both Sultr1;1 and Sultr1;2 in roots was strongly decreased upon exposure to sulfate and chloride salinity, but that of Sultr1;2 to a lesser extent by chloride salinity (Fig. 5). In the shoot, both Sultr1;1 and Sultr1;2 were hardly expressed and Sultr1;2 was not significantly affected by any of the salts while Sultr1;1 was decreased by both sulfate salts. The vacuolar sulfate transporters Sultr4;1 and 4;2 were differentially affected by the different salts in shoot and roots (Fig. 5). Sultr4;1, the transporter with the higher overall expression, was increased by the sulfate salts in both shoot and roots. Sultr4;2 was increased by sulfate salts in the shoot, however the effect appeared to be less sulfate specific, as KCl also significantly increased expression. No significant effects on Sultr4;2 in roots were observed. Relative expression of ATP-sulfurylase (ATPS) was not significantly affected by any of the salts, in the shoots or in roots. APS-reductase (APR), was decreased by sulfate salts in roots, but hardly changed in the shoot. Sulfite reductase (SiR) was significantly and similarly decreased by all salts in the shoot, but remained unaffected in roots (Fig. 5).

## Discussion

The present study revealed a higher toxicity of Na_2_SO_4_ over NaCl for seedlings of *B. rapa* L. (Fig. 1), as found previously in studies with other *Brassica* species (e.g. Paek et al. 1988). Na_2_SO_4_ was the only salt in the present study that caused a decrease in *Fv/fm* (Fig. 2), indicating photochemical stress. As stomatal resistance was increased by all salts similarly (Fig. 2) it may be concluded that differences in osmotic stress were responsible for differences in toxicity. In agreement with these results, Reginato et al. (2014) found a similar increase of osmotic potential in the shoot of *Prosopis strombulifera* plants exposed to NaCl and Na_2_SO_4_. The results of the present study show that *B. rapa* was relatively more sensitive to Na_2_SO_4_ salinity than other Brassica species (e.g. *B. carinata*; Canam et al. 2013).

There are two possible general explanations for the observed difference in toxicity. The first is an altered toxicity of sodium by the accompanying anion. A promotion of sodium toxicity by sulfate could, for example, be caused by an increased uptake or accumulation of sodium. An imbalanced cation-anion uptake may also be expected, as the plasma membrane is less permeable for sulfate than for chloride. The consequence may be a stronger negative effect on other cations, such as potassium or calcium, which experience a competition with sodium. The second explanation for the increased toxicity of Na_2_SO_4_ over NaCl is a higher toxicity of sulfate compared to chloride, either caused by a higher accumulation or a higher tissue toxicity of sulfate over chloride. In this scenario, the additive toxicities of sodium and sulfate would result in the toxicity of Na_2_SO_4_, rather than an interactive effect of sulfate with sodium. The comparison of sodium salts with potassium salts in the present study revealed that K_2_SO_4_ impaired growth whilst KCl had no significant effect (Fig. 1). Furthermore, sodium content was higher upon exposure to NaCl (Fig. 4; also found by Renault et al. 2001) and therefore sodium accumulation does not explain the higher toxicity of Na_2_SO_4_. In conclusion, the higher toxicity of Na_2_SO_4_ over NaCl was most likely due to the higher toxicity of sulfate over chloride, and not to a different sodium toxicity caused by the accompanying anion. The only indication for a synergistic negative effect of sodium and sulfate on photosynthetic efficiency was the observed decrease of Fv/fm in plants upon exposure to Na_2_SO_4_ (Fig. 2).

Sulfate contents in shoot and roots upon exposure to Na_2_SO_4_ and K_2_SO_4_ were increased relatively less than chloride upon exposure to NaCl and KCl based on absolute concentrations (Fig. 3). If, however, the divalency of sulfate is considered, the increases were relatively similar. The higher toxicity of the sulfate salts could therefore not be attributed to an imbalanced cation/anion uptake. It seems, however, that the translocation of chloride to the shoot was more effectively restricted upon exposure to NaCl than the translocation of sulfate upon exposure to Na_2_SO_4_. When the external concentration was doubled, chloride content in the shoot did not significantly increase further, whilst the sulfate content further increased and was close to the external sulfate concentration (Fig. 3). Sulfate content in the roots remained, in contrast, at the same level. This may indicate a difference in regulation of the translocation of chloride and sulfate to the shoot under saline conditions. The unrestricted translocation of sulfate to the shoot appears as a lack of a defense response against toxic concentrations of sulfate and could be one target for improvement by breeding.

There were slightly larger decreases found in the tissue contents of some nutrients caused by sulfate salts. Calcium is important for membrane integrity and function (Hepler 2005), signaling (Roberts and Harmon 1992) as well as the activity of certain enzymes (Kindt et al. 1980). Replacement of calcium from membranes and cell walls by sodium was often suggested to be one of the primary responses to salt stress (Cramer et al. 1985; Lynch et al. 1987; Rengel 1992). In the present study, all salts strongly decreased calcium content in shoot and roots. In the shoot, the decrease was stronger in plants exposed to sulfate salt. The relatively low solubility of Ca_2_SO_4_ could lead to an additional immobilization of calcium by internal crystallization, for example in the apoplast. Additional calcium has been shown to ameliorate salt stress and different possible mechanisms have been suggested, for example control of aquaporins (Carvajal et al. 2000), improved potassium homeostasis (Shabala et al. 2006) or increase of membrane permeability by additional calcium (Tuna et al. 2007). There are, however, no studies up to now on the specificity of this amelioration for stress caused by different salts.

The Group 1 sulfate transporters are responsible for the active uptake of sulfate uptake at the root plasma membrane (Smith et al. 1995). In the present study, similarly to previous observations in Brassica species (Buchner et al. 2004a; Koralewska et al. 2007, 2008, Koralewska et al. 2009; Reich et al. 2015), Sultr1;2 was the primary sulfate transporter expressed in the root, whereas expression of Sultr1;1 was very low. Despite the fact that the expression of both transporters in the roots was strongly down-regulated upon exposure to sulfate salts (Fig. 5) sulfate accumulated in the plants to toxic levels. The bulk of the sulfate influx under the high external sulfate concentrations was most likely passive influx, as hypothesized for chloride under saline conditions (White and Broadley 2001). Under these conditions, sulfate might pass the root plasma membrane via the remaining sulfate transporters, other anion-transporters with a minor affinity for sulfate and/or possibly via anion channels. Loading into the phloem and balancing sulfate fluxes between the vascular tissue and sink organs are assumed to be mediated by sulfate transporters of the Group 2, as well as Sultr1;3 (Buchner et al. 2004b) and Sultr3;5 (Kataoka et al. 2004a). In the shoot, both Group 1 sulfate transporter genes were hardly expressed (Fig. 5) and they probably play only a minor role for inter-organ sulfate fluxes under high sulfate conditions. Additionally, part of the fluxes may be mediated by channels, as found by Frachisse et al. (1999), who showed that voltage-dependent anion channels exist in hypocotyl cells of *Arabidopsis thaliana* which are not only highly permeable for, but also activated by, sulfate.

The decreased expression of the Group 1 sulfate transporters in the roots under salinity did not appear to be sulfate specific (Fig. 5). Chloride salts also decreased expression of the Sultr1;1 and 1;2 in roots, however, expression of Sultr1;2 was affected to a lower extent by chloride than by sulfate. These results suggest that the expression of sulfate transporters in roots was not only regulated by the sulfate level but at least partly also by the presence of other anions, such as chloride. Such a putative non-specific regulation of ion-specific transporters by the overall anion concentration or cation-anion balance should be studied further. The decrease of one anion in the plant tissue whilst another anion is supplied in excess could be caused not only by an electrochemical antagonisms but also by a control of the gene expression of ion-transporters. Alternatively, diminished demand for sulfate by impaired growth under salt stress may explain the down-regulation of the transporters.

The expression of the vacuolar sulfate transporters was measured as an indication for the effects of the different salts on sulfate storage and remobilization. Sultr4;1 and 4;2 are known to be responsible for the efflux of sulfate across the tonoplast into the cytosol under sulfur deprivation. The upregulation of the expression of these transporters under sulfur deficiency is well studied and most likely a strategy to remobilize sulfate from the vacuolar storage (Hawkesford 2000; Kataoka et al. 2004b). The relatively low abundance of transcripts of Sultr4;2 corresponds to other studies which showed that this transporter plays only a minor role under sulfur sufficient conditions (Buchner et al. 2004a; Kataoka et al. 2004b). In the present study, an excess of sulfate led to an increase of both, Sultr4;1 and 4;2 in the shoot and of Sultr4;1 in roots (Fig. 5). For Sultr4;1 the increase was mostly sulfate specific, with chloride salts only causing a minor, non-significant increase. Sultr4;2 was increased by chloride salts to the same extent as by sulfate salts; however, as mentioned above, the overall level of expression was relatively low. An increase in the transcript abundance of Sultr4;1 and 4;2 indicates an increased re-mobilization of sulfate from the vacuoles into the cytosol, from where it could reach the chloroplasts, the place it most likely led to the toxic effects observed upon exposure to Na_2_SO_4_ and K_2_SO_4_. One explanation for the observed up-regulation of the vacuolar sulfate transporters under an excess sulfate supply could be a control by the sulfate gradient across the tonoplast. Under optimal sulfate supply, cytosolic and vacuolar sulfate levels are very similar (Cram 1983). Under sulfur deficiency, as well as under sulfate excess, the cytosolic sulfate concentration drops relative to the sulfate concentration in the vacuole. Under deficiency, sulfate reduction exceeds sulfate import by Sultr1;1 and 1;2, causing a decrease in cytosolic sulfate concentration relative to the vacuole and leading to an upregulation of the vacuolar transporters to remobilize stored sulfate. Under sulfate excess, the incoming sulfate leads to a down-regulation of Sultr1;1 and 1;2, which probably has some protective effect against increased external sulfate, but does not avoid sulfate influx under prolonged exposure. Excess sulfate in the cytosol is immediately transported to the vacuole, following the inward proton gradient at the tonoplast (by a still unidentified, ATP-dependent mechanism which is thought to provide salt tolerance; Kaiser et al. 1989). Vacuolar sulfate concentrations increase relative to cytoplasmic concentrations until the sulfate gradient at the tonoplast might trigger an up-regulation of vacuolar transporters and to an efflux of sulfate into the cytosol. This is probably a protective mechanism to avoid an over-accumulation of sulfate in the vacuole (and maybe also excess acidification) under normal conditions and to ensure sulfate remobilization under deficiency Kataoka et al. (2004b)). Under sulfate excess it may be responsible for the detrimental efflux of sulfate from the vacuole into the cytosol.

An alternative explanation for the up-regulation of Sultr4;1 and 4;2 by sulfate excess is that they are bidirectional sulfate transporters and responsible for sulfate flux in both directions across the tonoplast, and not only for sulfate efflux from the vacuole. The accumulation of sulfate in the vacuole under normal conditions follows the charge gradient at the tonoplast (Martinoia et al. 2000) and is therefore presumably mainly determined by the ATP-driven translocation of protons. For this reason, sulfate remobilization into the cytosol has to be facilitated by active transport by Sultr4;1 and Sultr4;2 against the charge gradient. Because this process is relatively slow, sulfate remobilization from the vacuole is a potential bottleneck for sulfur use efficiency (Bell et al. 1995; Hawkesford 2000). Under sulfate excess, sulfate flux into the vacuole might at some point diminish the charge gradient that drives passive, charge-driven sulfate influx. This would make active translocation of sulfate into the vacuole necessary to avoid high sulfate concentrations in the cytosol and could be facilitated, for example, by Sultr4;1 and Sultr4;2. Bidirectional ion transport is suggested for vacuolar potassium transporters (Maathuis 2006) and some nitrate transporters (Léran et al. 2013), however, is generally very rare. Sultr4;1 and 4;2 might also be involved in the regulation of sulfate translocation from root to shoot, as indicated by the retention of sulfate in roots of *Arabidopsis thaliana* double knock-out mutants for these transporters (Kataoka et al. 2004b).

The three enzymes measured in the present study fulfill key steps in sulfate assimilation. ATP sulfurylase (ATPS) catalyzes the first step by activating sulfate to 5′-adenylylsulfate (APS). After that, APS is reduced by APS reductase (APR) to sulfite and subsequently reduced to sulfide by sulfite reductase (SiR; reviewed for example by Saito 2004). The regulation of the gene expression of these enzymes by sulfur deficiency and by the availability of reduced sulfur compounds is well studied, however, the exact signaling and the compounds involved are still under discussion. Sulfate is reduced in the chloroplasts (plastids in root), but the expression levels of the three sulfur assimilatory enzymes were very differently affected by the different salts. The expression of ATPS remained unaffected by all treatments in both, shoot and roots (Fig. 5). Similarly, transcript levels of ATPS in both shoot and roots of *B. rapa* were also hardly affected upon foliarly absorbed excess sulfur (H_2_S and SO_2_; Aghajanzadeh et al. 2016). Because of the relatively low affinity of this enzyme for sulfate, the in situ sulfate concentration in the chloroplast was suggested to play a key regulatory role in the rate of sulfate reduction (Stulen and De Kok 1993), however, the expression of the enzyme itself seems not be regulated by high sulfate or reduced sulfur compounds. APR is considered to be the key-regulating enzyme in the sulfur reduction pathway (Hell et al. 2002; Vauclare et al. 2002; Hawkesford and De Kok 2006). The expression of APR was not significantly increased by NaCl salinity, contrary to results obtained in *Arabidopsis thaliana* (Koprivova et al. 2008), but was decreased in roots upon sulfate salinity (Fig. 5). Exposure to an excess of Na_2_SO_4_ also leads to an increase in the thiol levels in shoot and roots (Reich et al. 2015), supporting the suggestion that the expression of APR is regulated by reduced sulfur compounds (Koralewska et al. 2008, 2009; Davidian and Kopriva 2010). Expression in the shoot, however, was not significantly affected. The expression of SiR was decreased by all salts in the shoot in an anion-unspecific manner (Fig. 5).

In summary, the present study shows that the increased toxicity of Na_2_SO_4_ over NaCl in *B. rapa* is due to the increased toxicity of sulfate over chloride rather than a promotion of sodium toxicity by sulfate. The increase of the expression of vacuolar sulfate transporters responsible for the efflux of sulfate from the vacuole is suggested to play a crucial mechanistic role for sulfate toxicity and a target for improvement via breeding. The upregulation of Sultr4;1 and 4;2 probably avoids excess sulfate accumulation in the vacuole but leads to a detrimental increase in cytosolic concentrations. Ancestors of the modern *Brassica* crops derive at least partly from maritime, salt-influenced habitats (Dixon 2007) and were probably moderately salt tolerant. This suggests that increased salt tolerance could be re-introduced into modern cultivars by breeding. Increasing the vacuolar storage capacity for sulfate, as well as a restriction of the translocation to the shoot are possible strategies to improve tolerance to excess sulfate levels and should be investigated in more detail. Sulfate exclusion could provide tolerance, but to date, no mechanisms for active sulfate export from root cells to the external medium have been described. Interactions of sulfate with other nutrients have been studied recently under sulfate deprivation and H_2_S fumigation (Reich et al. 2016) and the present study revealed interactions under sulfate excess. The stronger decrease of calcium, magnesium and manganese by sulfate salts could contribute to their increased toxicity compared to chloride salts. Specific amelioration of chloride and sulfate toxicity by these nutrients should be investigated in the future.
